# Association of personality traits with rumination improvement following cognitive behavioral therapy in major depression: an observational study

**DOI:** 10.3389/fpsyt.2025.1671393

**Published:** 2026-01-16

**Authors:** Sachiko Noda, Atsuo Nakagawa, Satoshi Umeda, Mizuki Amano, Waka Nogami, Sakae Ihara, Yuki Kobayashi, Mire Ozawa Kinouchi, Ryo Takemura, Hiroyuki Uchida, Nariko Katayama

**Affiliations:** 1 Department of Neuropsychiatry, Keio University School of Medicine, Tokyo, Japan; 2 Department of Neuropsychiatry, St. Marianna University School of Medicine, Kawasaki, Japan; 3 Department of Psychology, Faculty of Letters, Keio University, Tokyo, Japan; 4 Clinical and Translational Research Center, Keio University Hospital, Tokyo, Japan; 5 Health Center Mental Health Division, Keio University, Tokyo, Japan

**Keywords:** major depressive disorder, cognitive behavioral therapy, personality traits, rumination, brooding

## Abstract

**Introduction:**

Cognitive behavioral therapy (CBT) is effective for major depressive disorder (MDD), yet individual responses vary. Personality may relate to outcomes, but its role in brooding rumination during CBT remains unclear. This study tested whether baseline personality traits are associated with brooding change in patients with MDD receiving CBT.

**Methods:**

In this prospective observational cohort, 75 outpatients were allocated to CBT + treatment-as-usual (TAU) (n=33 baseline; n=30 longitudinal) or TAU alone (n=42; n=38) based on clinical course and preference. The Ruminative Responses Scale (RRS) brooding subscale and the 17-item Grid-Based Hamilton Depression Rating Scale (GRID-HAMD_17_) were assessed at baseline and 16 weeks; the Temperament and Personality Questionnaire (T&P) was assessed at baseline. Multiple linear regressions within each group examined associations between baseline traits and brooding change, adjusting for sex, baseline brooding, and baseline GRID-HAMD_17_. A trait-wise single-predictor sensitivity analysis used the same covariates.

**Results:**

Both groups demonstrated significant within-group improvements. Hedges’ g (95% CI): CBT—brooding 0.48 (0.11–0.85), GRID-HAMD_17_ 1.07 (0.63–1.50); TAU—brooding 0.84 (0.47–1.21), GRID-HAMD_17_ 1.46 (1.01–1.91). In CBT, higher anxious worrying was associated with greater brooding reduction (eight-predictor model: B = 0.71, *p* = 0.026) and remained significant in the single-predictor analysis (B = 0.36, *p* = 0.014). Self-criticism showed a negative association in the eight-predictor model (B=−0.60, *p* = 0.043) but did not persist in the single-predictor analysis. In TAU, no personality trait was associated with brooding change. Adjusted between-group differences in change (brooding, GRID-HAMD_17_, total DDD) were not significant.

**Limitations:**

Nonrandomized allocation, modest sample sizes, and two assessment time points limit precision and power; the eight-predictor model in CBT may be prone to overfitting, so findings are exploratory.

**Conclusion:**

In this observational cohort, personality traits, with anxious worrying appearing relevant, were associated with brooding change within CBT, whereas between-group differences were not significant. These hypothesis-generating results require validation in adequately powered randomized trials to inform stratified depression care.

## 1 Introduction

Major depressive disorder (MDD) is a prevalent and debilitating mental health condition with a substantial global impact and a leading cause of disability-adjusted life years (DALYs) in developed countries (1). In Japan, the burden is particularly notable, with a lifetime prevalence of 16.6% and substantial associated economic costs (2, 3). A defining feature of MDD is its recurrent and chronic course, which complicates long-term management and contributes to declining recovery rates—approximately 60% at two years and reduced to around 30% by six years—despite therapeutic efforts (4, 5). Therefore, sustained recovery of MDD is a critical and unmet need in the treatment.

As an established and evidence-based treatment of MDD, cognitive behavioral therapy (CBT) offers equivalent efficacy to contemporary antidepressant medications (6) and is often the preferred modality for patients (7). Its clinical utility is further supported by the evidence from the UK and Japan (8–10) demonstrating long-term sustained benefits, particularly when used adjunctively for treatment-resistant depression, where remission rates significantly improve and persist for 12–40 months post-intervention. This body of evidence underpins the recommendations in major clinical practice guidelines (11–13) advocating CBT implementation, adjusted for severity, in managing MDD.

Understanding the cognitive mechanisms underlying the persistence and recurrence of MDD is crucial, with particular attention to depressive rumination. This symptom involves repetitive focus on the self and negative affect, contributing to the prolongation of depressive states (14). The brooding, a rumination component, characterized by its self-evaluative and nonconstructive nature, differs clinically from reflective pondering, which is more adaptive (15). Brooding is associated with symptom exacerbation and chronicity, suggesting its relevance as a potential target for CBT (16, 17). Furthermore, accumulating evidence supports the hypothesis that rumination reduction partially mediates the therapeutic effects of CBT in individuals with depression (18). Meta-analytic evidence, including a recent transdiagnostic review, indicate that CBT yields moderate reductions in rumination—with larger effects when protocols explicitly target rumination—and that decreases in rumination co-occur with improvements in depressive symptoms (19, 20).

In addition to cognitive factors, such as rumination, individual personality traits are recognized as influences on MDD treatment outcomes. Higher levels of neuroticism and lower extraversion have been consistently associated with poorer clinical courses, including chronicity, relapse, and diminished treatment response (21–23). Our previous prospective study highlighted the role of personal reserve trait, a specific facet of low extraversion, in predicting remission maintenance and treatment success (24, 25). While existing cross-sectional studies have demonstrated associations between rumination and personality traits (26, 27), prospective studies examining how personality influences rumination during psychological interventions such as CBT remain limited. Investigating the role of personality characteristics in CBT response may offer valuable insights for tailoring treatment approaches, particularly given the established link between rumination and the recurrence and chronicity of MDD.

Addressing the role of individual differences in CBT outcomes remains an essential yet incompletely understood area in CBT research (28). This study aimed to clarify the influence of specific personality traits on the reduction of brooding, a maladaptive component of rumination, in patients undergoing CBT for MDD. We tested the hypothesis that certain baseline personality traits, measured using the Temperament and Personality Questionnaire (T&P) (29), are associated with changes in brooding scores on the Ruminative Responses Scale (RRS) (15) over a 16-week treatment period. We included a treatment-as-usual (TAU) group for comparison to assess whether these associations were specific to CBT. By examining these longitudinal associations, this study may contribute to growing evidence of personalized approaches to CBT for MDD.

## 2 Materials and methods

### 2.1 Study design and setting

This prospective, observational cohort study was conducted between May 2020 and September 2024. It investigated how baseline personality traits impact improvements in brooding rumination in patients with MDD. This study followed the Strengthening the Reporting of Observational Studies in Epidemiology (STROBE) guidelines (30). Participants were recruited from four healthcare facilities in Japan that are involved in the treatment of mood disorders: one university hospital, two psychiatric hospitals, and one psychiatric clinic.

### 2.2 Ethical considerations

The study was approved by the Ethical Review Committee of the Keio University School of Medicine (approval number: 20200088) and was performed in accordance with the ethical standards of the Declaration of Helsinki. Informed consent was obtained from all the participants after explaining the study details, including aims and methods, verbally and through written documentation.

### 2.3 Participants

The eligibility criteria for enrollment were as follows: 1) a primary diagnosis of MDD confirmed according to the Diagnostic and Statistical Manual of Mental Disorders, Fourth Edition, Text Revision (DSM-IV-TR) (31) or Fifth Edition (DSM-5) (32) using the Structured Clinical Interview for DSM (SCID) administered by trained psychiatrists and 2) age between 20 and 75 years at the time of enrollment. The exclusion criteria were: 1) presence of a severe or unstable comorbid physical illness that could interfere with participation; 2) acute psychiatric symptoms presenting an imminent risk of self-harm or harm to others; and 3) unsuitability for participation determined by the attending physician or principal investigator. Potential participants were identified by the treating clinicians during routine clinical practice at the four sites. The clinicians provided a brief overview of the study to the potentially eligible patients. Those interested were scheduled for a separate dedicated session with the research staff for a comprehensive explanation of the study procedures and objectives, following which informed consent was obtained. Consistent with the observational design and with the aim of maximizing representativeness, all eligible patients who provided consent during the recruitment period were enrolled.

### 2.4 Procedures

#### 2.4.1 Treatment Groups

The participants were categorized into two groups based on their treatment course and preferences: CBT group, comprising patients who received CBT in addition to TAU, and TAU group, comprising patients who received only TAU. This categorization was non-randomized and reflected real-world clinical pathways.

##### 2.4.1.1 CBT group

Patients in the CBT group received 16 weekly 50-minute face-to-face CBT sessions, adhering to the structured protocol outlined in the CBT Therapist Manual for Depression by the Japanese Ministry of Health, Labor and Welfare (33). This manual is adapted from Beck’s original cognitive therapy model to suit Japanese cultural contexts and comprises six core components: 1) psychoeducation and assessment; 2) case formulation, problem clarification, and behavioral activation; 3) moods and automatic thought identification; 4) examining automatic thoughts and problem-solving; 5) core belief identification; and 6) summary and relapse prevention. Each session began with a collaborative agenda-setting, followed by a therapeutic discussion, and concluded by assigning action plans for patients to practice skills between sessions. Up to four additional CBT sessions were permitted, if deemed necessary by the therapist. A team of three psychiatrists and six clinical psychologists, all having master’s or doctoral degrees, provided the CBT. All therapists completed a 2-day intensive workshop and received ongoing weekly 1-hour group supervision, including case reviews and feedback, from an experienced CBT supervisor to ensure manual adherence. The CBT group received concurrent pharmacotherapy managed by their psychiatrists.

##### 2.4.1.2 TAU group

Participants in the TAU group received standard care for MDD, primarily pharmacotherapy, which was administered according to the clinical practice guidelines established by the Japanese Society of Mood Disorders. While supportive psychotherapy and lifestyle guidance were provided by psychiatrists as part of routine care, structured CBT was not administered.

##### 2.4.1.3 Pharmacotherapy management

Medication management for both groups followed the standard Japanese clinical guidelines for depression. Psychiatrists monitored the side effects and provided adherence support. No specific restrictions beyond standard clinical practice were imposed on the type or dosage of the prescribed psychotropic medications.

#### 2.4.2 Assessment procedures

At baseline, background information was obtained and a 17-item Grid-Based Hamilton Depression Rating Scale (GRID-HAMD_17_) (34) assessment was conducted. The participants completed self-reported RRS and T&P questionnaires. The RRS and GRID-HAMD_17_ assessments were repeated at the 16-week follow-up visit.

### 2.5 Measures

#### 2.5.1 Rumination (RRS)

The brooding subscale of the Japanese version of the RRS was used to assess maladaptive rumination. This subscale consists of five items rated on a 4-point Likert scale (1 = almost none to 4 = almost always), assessing the tendency to compare one’s current situation with an unachieved standard. Higher scores indicate greater brooding rumination. It was chosen because it specifically captures the rumination component that is strongly linked to depressive symptoms and treatment outcomes (15). The Japanese RRS has established reliability and validity, with a Cronbach’s alpha of 0.77 for the brooding subscale (35).

#### 2.5.2 Depressive symptom severity (GRID-HAMD_17_)

The GRID-HAMD_17_ Japanese version (36) was used to assess the severity of depressive symptoms over the past 7 days. This observer-rated scale evaluates symptoms based on both the frequency and intensity dimensions, with total scores ranging from 0 to 52. Higher scores indicated greater symptom severity. All GRID-HAMD_17_ assessments were conducted by trained raters (psychiatrists or clinical psychologists) who demonstrated excellent inter-rater reliability (intraclass correlation coefficient [ICC] = 0.94–0.98) following extensive training.

#### 2.5.3 Personality traits (T&P)

The Japanese version of the T&P (37) was used to evaluate eight personality traits relevant to non-melancholic depression. The T&P potentially informs treatment selection (38) and predicts treatment outcomes in depression (39, 40). This 109-item self-report questionnaire measures the following personality dimensions on a 4-point Likert scale: anxious worrying, self-criticism, rejection sensitivity, irritability, self-centeredness, perfectionism, private self-consciousness, and social avoidance. Higher scores reflect stronger tendencies for each trait. The T&P factors represent clinically relevant facets derived from the broader dimensions of the Five-Factor Model (FFM) of personality (41), offering a clinically applicable assessment framework for personality traits. The validity and reliability of the Japanese version have been confirmed (37).

#### 2.5.4 Background information

Demographic characteristics (age, sex, employment status, marital status, and living situation) and clinical information (cumulative duration of depressive episodes, duration of the current episode, history of suicide attempts, and family history of psychiatric disorders) were collected at baseline through clinical interviews and medical record review.

### 2.6 Statistical analysis

All statistical analyses were performed using IBM SPSS Statistics for Mac, version 29.0.2.0 (IBM Corp., Armonk, NY). A two-sided *p*-value less than 0.05 was considered statistically significant. The analyses were conducted using the complete case data, excluding cases with missing values for the variables involved in each specific analysis.

#### 2.6.1 Baseline comparisons

To examine the baseline differences between the CBT and TAU groups, independent sample t-tests and chi-square test were used for continuous (demographic, clinical, and psychological scale scores) and categorical variables, respectively.

#### 2.6.2 Within-group changes

Paired t-tests were used to evaluate within-group (CBT and TAU) changes from baseline to the 16-week follow-up in the primary (RRS brooding score) and secondary outcomes (GRID-HAMD_17_ score). Changes in medication dosage, calculated separately as defined daily doses (DDD) for antidepressants and antipsychotics, were also assessed within each group. All within-group analyses were performed on paired cases, and Hedges’ g for dependent observations (small-sample corrected) were reported with 95% confidence intervals (CIs).

#### 2.6.3 Between-group changes

To test whether overall changes differed by treatment group, we conducted analysis of covariance (ANCOVA) for 16-week outcomes (RRS brooding, GRID-HAMD_17_, total DDD). Each model included treatment group (CBT vs TAU) as a fixed factor and baseline score and sex as covariates. We report estimated marginal means (EMMs), adjusted group differences (CBT–TAU) with 95% CIs, *p*-values, and partial η² as an effect-size index.

#### 2.6.4 Cross-sectional correlations

We computed two-sided Pearson correlations between each personality trait and RRS brooding at baseline and 16 weeks (**Supplementary Tables S1A, B**), and between each trait and GRID-HAMD_17_ at baseline and 16 weeks (**Supplementary Tables S1C, D**). Correlations are reported overall and within CBT and TAU. Benjamini–Hochberg FDR was noted within each family of eight tests per stratum.

#### 2.6.5 Factors associated with change in brooding scores

To explore potential predictors of change in brooding scores, multiple linear regression analyses were conducted separately for the CBT and TAU groups. The dependent variable was the RRS brooding score change, calculated as the baseline score minus the 16-week follow-up score. The eight T&P factor scores at baseline were included as the independent variables. Multicollinearity diagnostics were performed before interpreting the regression models. We examined variance inflation factors (VIFs) as an index of collinearity and report the maximum VIF per model. To account for potential confounding effects, sex (which differed significantly between groups at baseline), baseline GRID-HAMD_17_ scores, and RRS brooding scores, which are clinically relevant to both depression severity and rumination, were included as covariates in both models.

In addition to the main eight-predictor models, we conducted trait-wise adjusted sensitivity analyses. Each personality trait was entered in a separate linear model with the same covariates (sex, baseline brooding, baseline GRID-HAMD_17_). We reported unstandardized B, 95% CIs, and *p*-values. We evaluated model assumptions for all linear models: residual normality (Q–Q plots; Shapiro–Wilk), multicollinearity (VIFs), and influence (Cook’s distance).

## 3 Results

### 3.1 Patient demographics and baseline characteristics

A total of 75 participants were enrolled and assessed at baseline. Of them, 68 (90.7%) provided sufficient data for the 16-week follow-up assessment and were included in the longitudinal analyses. Seven participants (three from the CBT group and four from the TAU group) were excluded from the analysis due to missing data in one or more follow-up measures. Thus, baseline analyses included 75 participants (CBT, n = 33; TAU, n = 42), whereas longitudinal analyses included 68 participants (CBT, n = 30; TAU, n = 38).

The baseline demographic, clinical, and psychological characteristics of the participants are shown in **Table 1**. On average, they were in their mid-to-late 30s. The proportion of women was significantly higher in the CBT group (72.7%) than in the TAU group (47.6%; χ² = 4.84, *p* = 0.028). The groups did not differ significantly at baseline in working status, marital status, cohabitation status, duration of depressive episodes (lifetime or current), history of suicide attempts, or family history of psychiatric disorders. Furthermore, no significant baseline differences were observed between the groups in any T&P personality trait score, RRS brooding score, or GRID-HAMD_17_ score (all *p* > 0.05).

**Table 1:** Participant characteristics at baseline.

Characteristics	CBT (n = 33)	TAU (n = 42)	*p*-value
Demographic characteristics			
	Mean (SD)	Mean (SD)	
Age (years)	34.3 (11.9)	39.3(11.6)	0.07
	N (%)	N (%)	
Female	24 (72.7%)	20 (47.6%)	0.028*
Working status			0.55
Employed, housewife, or student	14 (42.4%)	15(35.7%)	
Others (unemployed and on sick leave)	19 (57.6%)	27 (64.3%)	
Marital status			0.26
Married	13 (39.4%)	22 (52.4%)	
Single, divorced, or widowed	20 (60.6%)	20 (47.6%)	
Cohabiting	25 (75.8%)	35 (83.3%)	0.41
Clinical characteristics			
	Mean (SD)	Mean (SD)	
Lifetime duration of depressive episodes (months)	54.9 (75.7)	47.6 (67.52)	0.66
Duration of current depressive episode (months)	17.0 (20.7)	12.7 (27.8)	0.46
	N (%)	N (%)	
History of suicide attempts	5 (15.2%)	3 (7.1%)	0.26
Family history of psychiatric disorders	13 (39.4%)	14 (33.3%)	0.587
Psychological Assessment	Mean (SD)	Mean (SD)	*p*-value
anxious worrying	15.5 (4.9)	13.6 (5.3)	0.13
Personal reserve	11.5 (6.5)	14.0 (5.2)	0.67
Perfectionism	15.0 (5.1)	14.0 (5.2)	0.41
Irritability	8.5 (6.5)	10.1 (5.0)	0.23
Social avoidance	14.3 (5.9)	14.1 (5.0)	0.90
Rejection sensitivity	9.8 (5.7)	8.5 (5.6)	0.32
Self-criticism	16.3 (4.1)	15.5 (3.8)	0.39
Self-focused	3.3 (3.5)	4.2 (2.8)	0.24
RRS brooding score	15.1 (3.4)	13.6 (3.8)	0.097
GRID-HAMD_17_	16.7 (3.1)	17.9 (5.0)	0.24

*p-values were calculated using independent sample t-tests for continuous variables and chi-square tests for categorical variables. *p* < 0.05.

CBT, cognitive behavioral therapy; TAU, treatment as usual; SD, standard deviation; DDD, defined daily dose; T&P, temperament and personality; RRS, Ruminative Responses Scale; GRID-HAMD_17_, GRID version of the 17-item Hamilton Depression Rating Scale

#### 3.1.1 Within-group changes in rumination, depressive symptoms, and medication dosage

The changes in outcomes from baseline to the 16-week follow-up in each group are shown in **Table 2**. Both groups demonstrated statistically significant reductions in RRS brooding scores (CBT: mean change = 1.84, *p* = 0.01; TAU: mean change = 3.16, *p* < 0.001) and GRID-HAMD_17_ scores (CBT: mean change = 8.43, *p* < 0.001; TAU: mean change = 10.47, *p* < 0.001).

**Table 2:** Within-group changes in depression, rumination, and DDD from baseline to 16 weeks.

Group	Measure	Baseline mean (SD)	16-week mean (SD)	*p*-value	Hedges’ g [95% CI]
CBT N = 30	RRS brooding	15.07 (3.6)	13.23 (4.4)	0.01	0.48 [0.11–0.85]
	GRID-HAMD_17_	16.77 (2.6)	8.97 (6.5)	< 0.001	1.07 [0.63, 1.50]
	DDD (antidepressants)	1.0 (0.7)	1.0 (0.8)	0.87	
	DDD (antipsychotics)	0.04 (0.1)	0.06 (0.1)	0.30	
TAU N = 38	RRS brooding	13.45 (3.9)	10.29 (4.1)	< 0.001	0.84 [0.47, 1.2]
	GRID-HAMD_17_	17.67 (5.1)	7.26 (5.6)	< 0.001	1.46 [1.01, 1.9]
	DDD (antidepressants)	0.87 (0.8)	1.1 (0.9)	0.062	
	DDD (antipsychotics)	0.06 (0.2)	0.06 (0.1)	0.85	

Baseline/16-week means and SDs are computed on paired cases only (participants with both time points). Mean change is the paired mean difference (baseline − week 16). Effect sizes denote Hedges’ g for dependent observations with 95% CIs. Positive change indicates a reduction from baseline to Week 16.

CBT, cognitive behavioral therapy; TAU, treatment as usual; RRS brooding, Ruminative Responses Scale brooding subscale; GRID-HAMD_17_, GRID version of the 17-item Hamilton Depression Rating Scale; DDD, defined daily dose.

There was no significant change in the mean DDD of antidepressants (*p* = 0.87) or antipsychotics (*p* = 0.30) between baseline and follow-up in the CBT group. In the TAU group, mean DDD of antidepressants showed an increasing trend, which was not statistically significant (*p* = 0.062). There was no significant change in the mean antipsychotic DDD in the TAU group (*p* = 0.85). Within-group effect sizes (Hedges’ g) were CBT—brooding 0.48 (95% CI 0.11–0.85), GRID-HAMD_17_ 1.07 (0.63–1.50), TAU—brooding 0.84 (0.47–1.21), and GRID-HAMD_17_ 1.46 (1.01–1.91) (**Table 2**).

#### 3.1.2 Between-group changes in rumination, depressive symptoms, and medication dosage

Adjusted between-group differences were not statistically significant for RRS brooding (CBT–TAU = +1.71, 95% CI −0.07 to 3.50, *p* = 0.060, partial η² = 0.054), GRID-HAMD_17_ (+1.36, −1.68 to 4.40, *p* = 0.38, partial η² = 0.012), or total DDD (−0.20, −0.53 to 0.13, *p* = 0.24, partial η² = 0.020). Thus, overall changes in brooding, depressive symptoms, and medication dose were comparable between groups after baseline adjustment (**Table 3**).

**Table 3:** Between-group changes in depression, rumination, and DDD from baseline to week 16.

Outcome	EMM: TAU [95% CI]	EMM: CBT [95% CI]	CBT−TAU [95% CI]	*p*	η^2^
RRS brooding	10.83 [9.68 to 11.99]	12.55 [11.24 to 13.85]	+1.71 [−0.07 to 3.50]	0.06	0.054
GRID-HAMD_17_	7.41 [5.44 to 9.38]	8.77 [6.55 to 10.99]	+1.36 [−1.68 to 4.40]	0.38	0.012
Total DDD	1.32 [1.11 to 1.53]	1.12 [0.87 to 1.37]	−0.20 [−0.53 to 0.13]	0.24	0.02

ANCOVA models used 16-week scores as outcomes (group as fixed factor) and the following covariates: RRS brooding—baseline brooding, sex; GRID-HAMD_17_—baseline GRID-HAMD_17_, sex; and Total DDD—baseline total DDD, sex. We report estimated marginal means (EMM) and adjusted mean differences (CBT − usual care) with 95% CIs; tests are two-sided (α =0.05); effect size is partial eta-squared(η^2^). Model checks: Levene’s *p* = 0.521 (brooding), 0.481 (GRID-HAMD_17_), 0.883 (DDD). Group-effect F statistics: F (1,64) =3.68 (brooding), F (1,66) =0.80 (GRID-HAMD17), F (1,70) =1.42 (DDD).

CBT, cognitive behavioral therapy; TAU, treatment as usual; RRS, Ruminative Responses Scale; GRID-HAMD_17_, GRID version of the 17-item Hamilton Depression Rating Scale; DDD, defined daily dose; CI, confidence interval.

### 3.2 Cross-sectional correlations at baseline and week 16

At baseline, brooding showed moderate correlations with several traits in both groups, most consistently rejection sensitivity and self-criticism, with additional signals for anxious worrying and personal reserve (**Supplementary Table S1A**). At 16 weeks, cross-sectional correlations with brooding were attenuated; none remained significant after FDR in CBT, whereas rejection sensitivity was largest in TAU (**Supplementary Table S1B**). For GRID-HAMD17, correlations with traits were small at baseline and 16 weeks and did not survive FDR (**Supplementary Tables S1C, D**). Change-based associations are presented in the main text (**Tables 4**, **5**).

**Table 4:** Association between T&P factors and brooding change in CBT and TAU—eight-predictor models.

Variable	CBT (N = 30)			TAU (N = 38)		
T&P	B	95% CI	*p*-value	B	95% CI	*p*-value
Anxious worrying	0.71	[0.09, 1.3]	0.026*	0.38	[–0.33, 0.41]	0.83
Personal reserve	0.30	[–0.09, 0.68]	0.12	-0.03	[–0.48, 0.43]	0.90
Rejection sensitivity	0.10	[–0.24, 0.43]	0.57	0.002	[–0.40, 0.40]	0.99
Perfectionism	0.03	[–0.41, 0.47]	0.89	0.05	[–0.27, 0.37]	0.74
Self-focused	-0.14	[–0.90, 0.51]	0.57	0.08	[–0.50, 0.66]	0.79
Social avoidance	-0.20	[–0.62, 0.23]	0.34	-0.09	[–0.58, 0.40]	0.70
Irritability	-0.21	[–063, 0.21]	0.31	-0.004	[–0.37, 0.36]	0.98
Self-criticism	-0.60	[–1.1, –0.02]	0.043*	-0.05	[–0.63, 0.53]	0.86

Regression coefficients (B), 95% CIs, and *p*-values are shown for multiple linear regression models predicting the change in RRS Brooding scores from baseline to week 16. CBT model: N = 30, R² = 0.498; TAU model: N = 38, R² = 0.314. *p < 0.05.

CBT, cognitive behavioral therapy; TAU, treatment as usual; T&P, temperament and personality; CI, confidence interval.

**Table 5:** Association between T&P factors and brooding change in CBT and TAU—single-predictor models.

Variable	CBT (N = 30)			TAU (N = 38)		
T&P	B	95% CI	*p*-value	B	95% CI	*p*-value
Anxious worrying	0.36	[0.08, 0.65]	0.014*	0.023	[–0.22, 0.27]	0.85
Personal reserve	0.22	[–0.038, 0.47]	0.093	-0.062	[–0.28, 0.15]	0.56
Rejection sensitivity	0.21	[–0.065, 0.49]	0.13	-0.026	[–0.31, 0.26]	0.85
Perfectionism	0.23	[–0.059, 0.53]	0.11	0.075	[–0.14, 0.29]	0.49
Self-focused	0.39	[–0.39, 0.47]	0.85	0.093	[–0.31, 0.50]	0.64
Social avoidance	-0.11	[–0.17, 0.40]	0.43	-0.15	[–0.37, 0.068]	0.17
Irritability	0.13	[–0.12, 0.37]	0.30	0.020	[–0.24, 0.28]	0.88
Self-criticism	0.11	[–0.28, 0.50]	0.58	-0.13	[–0.45, 0.19]	0.40

Covariates in all models: sex, baseline brooding, and baseline GRID-HAMD_17_. Regression coefficients (B), 95% CI, and p-values are shown. Each row comes from a separate model, including one personality trait (entered alone) plus the covariates (sex, baseline brooding, and baseline GRID-HAMD_17_). CBT, cognitive behavioral therapy; TAU, treatment as usual; T&P, temperament and personality; CI, confidence interval.

### 3.3 Factors associated with change in brooding scores

Multiple linear regression analyses were performed separately for the CBT and TAU groups to examine the association between baseline personality traits (T&P factors) and the magnitude of change in RRS-brooding scores over 16 weeks, after adjusting for baseline GRID-HAMD_17_ scores, baseline RRS-brooding scores, and sex (**Table 4**). Collinearity showed a mild elevation in the CBT eight-predictor model (max VIF = 5.54) and was lower in TAU (max VIF = 4.83); coefficients in the CBT model were interpreted with appropriate caution. In the single-trait models, all VIFs were <2.

**Figure 1:**
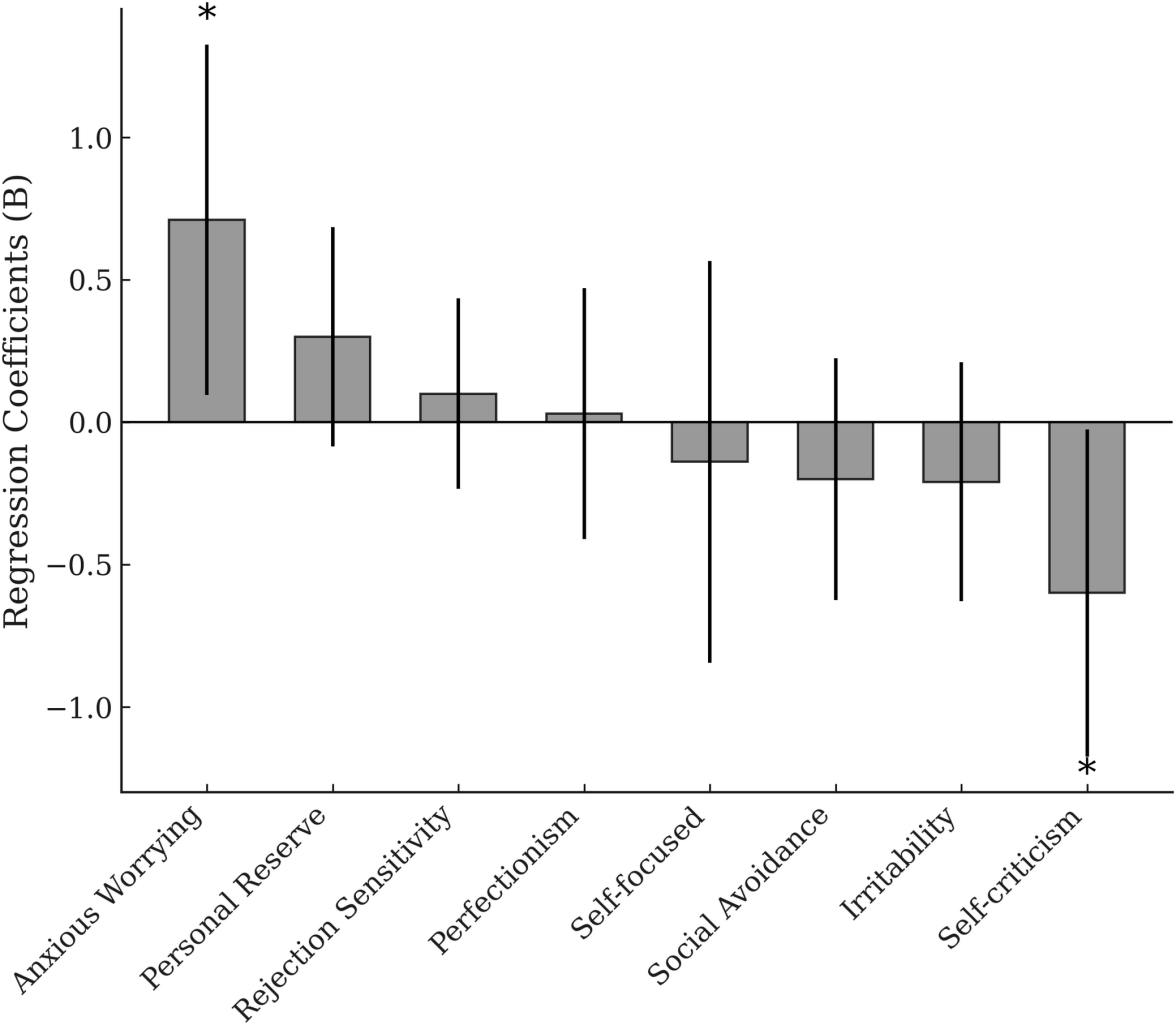
Associations between T&P factors and change in brooding in the CBT group (eight-predictor model). Bars show unstandardized regression coefficients (B) from the multiple linear regression adjusted for sex, baseline RRS brooding, and baseline GRID-HAMD_17;_ error bars indicate 95% CIs. Positive B indicates a greater reduction in brooding (change = baseline − week 16). Anxious worrying was associated with greater improvement (B = 0.71, 95% CI 0.09–1.32, *p* = 0.026), whereas self-criticism was associated with less improvement (B = −0.60, 95% CI −1.17 to −0.02, *p* = 0.043). *p < 0.05 (two-sided). T&P, temperament and personality; CBT, cognitive behavioral therapy; CI, confidence interval.

The regression coefficients for the CBT group are shown in **Figure 1**. In the CBT group, the regression model explained a significant portion of the variance in brooding score changes (Model R² = 0.498). Baseline anxious worrying was associated with greater improvement in brooding (B = 0.71, 95% confidence interval [CI] [0.09, 1.32], *p* = 0.026), while baseline self-criticism was associated with less improvement (B = -0.60, 95% CI [-1.17, -0.02], *p* = 0.043). No other T&P factors showed significant links in the CBT group (all *p* > 0.05). Covariates were not associated with changes in brooding score in this model.

In the TAU group, the overall regression model did not explain a significant variance (Model R² = 0.314). None of the baseline T&P personality traits were significantly related to the change in RRS-brooding scores (all *p* > 0.05). Similarly, the covariates were not significantly associated with brooding score in the TAU model.

According to trait-wise adjusted analyses (**Table 5**), anxious worrying in the CBT group continued to be associated with greater brooding reduction (B = 0.364, 95% CI 0.081–0.647, p=0.014), whereas all other traits were not significant (all p>0.05). In the TAU group, no trait exhibited a significant association with brooding change (all p>0.05).

Assumption checks supported model adequacy: residual Q–Q plots were approximately linear, and Shapiro–Wilk tests on residuals were non-significant in both groups (CBT: *p* = 0.221; TAU: *p* = 0.207).

## 4 Discussion

This study examined whether personality traits relate to treatment-related changes in brooding among patients with MDD. In the CBT group, higher baseline anxious worrying consistently predicted greater brooding reduction, whereas no personality predictors emerged in TAU. The self-criticism signal in the full model did not persist in single-predictor analyses and was treated as provisional. Because adjusted between-group differences did not reach significance for brooding, depression severity, or medication dose, these findings are most consistent with personality–brooding associations that emerge within the CBT context instead of group-level superiority of CBT over TAU; however, the modest sample size warrants cautious interpretation.

A notable finding was the positive association between higher baseline anxious worrying and a greater reduction in brooding in the CBT group. This personality trait, as measured by T&P, involves cognitive styles with pessimistic thinking and anticipatory anxiety tendencies when stressed, which may contribute to brooding (29). The cognitive restructuring strategies central to CBT, particularly those targeting maladaptive anticipatory thinking styles, may align well with the cognitive vulnerabilities observed in individuals with high anxious worrying. In line with this, our previous studies have indicated that CBT modifies brain activity and behaviors associated with future-oriented cognition (42, 43). These observations are consistent with CBT engaging processes relevant to brooding in some patients, particularly those with higher anxious worrying within the CBT group; however, CBT did not demonstrate a significant advantage over TAU at the group level.

By contrast, the signal for self-criticism was weaker and inconsistent across models. Self-criticism—persistent negative self-evaluation—may sustain brooding through rigid negative self-schemas and can complicate engagement with core CBT processes (e.g., collaborative work, homework adherence). Therefore, in this dataset, we regard self-criticism as a hypothesis-generating consideration instead of a robust predictor. Future work should include session-level measures (e.g., brooding trajectories, homework completion, therapeutic alliance) to clarify whether personality shapes engagement with CBT processes that mediate change.

A noteworthy distinction was observed in the TAU group, in which personality traits had no significant influence on brooding reduction. This finding contrasts with those of the CBT group, compatible with psychotropic medications acting through pathways that may differ from CBT, which can make outcomes less contingent on baseline personality traits (28, 44, 45). In Japanese clinical practice, CBT is often recommended for patients with mild-to-moderate MDD, especially following an inadequate response or intolerance to pharmacotherapy or based on patient preference (13). Therefore, our results suggest a strategic implication relevant to optimizing treatment selection. When considering CBT after an initial non-response to medication, a pre-CBT assessment of relevant personality traits could be valuable for tailoring the therapeutic approach and maximizing the potential benefits of CBT for individual patients.

Because adjusted between-group differences did not reach significance for brooding, depression severity, or medication dose, group-level change possibly does not account for the observed personality–brooding associations. Instead, these associations appear to emerge within the CBT context. The concentration of trait–outcome associations on brooding change during CBT, combined with limited cross-sectional relationships at week-16 and the absence of FDR-significant associations for depressive symptoms, support a treatment-specific interpretation. Personality traits—particularly anxious worrying—may indicate the responsiveness of brooding to CBT instead of general post-treatment symptom levels. Considering the number of tests and the modest sample size, these findings should be interpreted cautiously and warrant replication in larger samples.

Several factors may help explain why CBT did not outperform TAU in this cohort. First, the study was observational with nonrandom treatment allocation. Despite adjustment for baseline brooding, baseline GRID-HAMD_17_, and sex, unmeasured case-mix differences and residual confounding may have attenuated between-group contrasts. In Japanese clinical settings, access to CBT remains limited and CBT is not routinely first-line; referrals often occur after suboptimal pharmacotherapy response or when clinical complexity raises concern about improvement under usual care. Such referral patterns could have preferentially channeled more refractory or complex cases into the CBT arm, resulting in biased comparisons preventing the detection of an advantage for CBT. Second, TAU in this study consisted of routine outpatient psychiatric care—medication management during regular clinic visits—delivered by board-certified psychiatrists; approximately all possessed formal CBT training, and included the supervisor for the CBT arm. TAU did not follow a CBT manual or include structured modules or homework; however, clinician expertise might have introduced occasional CBT-consistent elements, plausibly elevating TAU effectiveness and narrowing the incremental contrast with protocolized CBT. Additionally, CBT’s advantages are often expected to emerge in maintenance and relapse prevention; our 16-week horizon may have been too short to detect durability differences. Third, with two time points and modest sample sizes, the ANCOVA may have been underpowered to enable detection of small to moderate adjusted differences; the 95% CI for brooding (−0.07 to 3.50) is compatible with anything from no difference to a small advantage. Finally, regression to the mean and expectancy and/or other non-specific therapeutic factors possibly contributed to improvements in both groups.

### 4.1 Limitations

This observational, nonrandomized design is vulnerable to selection bias and residual confounding despite adjustment for baseline brooding, baseline GRID-HAMD_17_, and sex. In addition, sample sizes were modest with only two assessment points, limiting power for between-group contrasts and small effects. The eight-predictor regression in the CBT group may be prone to overfitting; although assumption checks were acceptable, estimates should be interpreted cautiously and validated independently. We also did not conduct a formal personality-trait × treatment interaction test, so within-CBT associations are not definitive evidence of moderation. Finally, medication management changed during follow-up (particularly in TAU), which may have influenced outcomes independently of psychotherapy.

### 4.2 Future directions

Randomized or stratified designs with prespecified personality trait-by-treatment interaction tests and adequate power are needed to determine whether personality traits moderate the change in brooding under CBT. Prospective work incorporating session-level assessments of brooding and GRID-HAMD_17_ together with session-level process measures (e.g., homework completion, use of cognitive strategies, therapeutic alliance) and longer post-treatment follow-up would permit trajectory and mediation analyses (e.g., mixed-effects models) and clarify whether personality relates to engagement with CBT mechanisms of change. External validation in independent cohorts will be important to confirm whether anxious worrying reliably marks brooding responsiveness to CBT, and targeted augmentation for self-critical thinking could be tested in hypothesis-driven trials.

In conclusion, in this observational cohort, personality traits, with anxious worrying appearing relevant, were associated with brooding change within CBT, whereas between-group differences were not significant. These hypothesis-generating results require validation in adequately powered randomized trials to inform stratified depression care.

## Data Availability

The raw data supporting the conclusions of this article will be made available by the authors, without undue reservation.
