# Supplementary material for: Association of personality traits with rumination improvement following cognitive behavioral therapy in major depression: an observational study
Source: Front Psychiatry. 2026 Jan 16;16:1671393. doi: 10.3389/fpsyt.2025.1671393 (PMC12855471; doi:10.3389/fpsyt.2025.1671393)
Supplement: Supplementary file 1 [file DataSheet1.pdf]

Supplementary Table S1: Correlations analyses between personality traits and symptom measures at baseline and 16 weeks

General note for Supplementary Tables S1A–S1D.
Two-sided Pearson correlation coefficients (r) are reported for the overall sample and separately by group (CBT, TAU). For each stratum (ALL, CBT, TAU), Benjamini–Hochberg false-discovery rate (q) corrections were applied within the family of eight tests. No covariates were included.

Abbreviations (for S1A–S1D).
CBT, cognitive behavioral therapy; TAU, treatment as usual; RRS brooding, Ruminative Responses Scale brooding subscale; GRID-HAMD17, GRID version of the 17-item Hamilton Depression Rating Scale; FDR, false-discovery rate.

S1A: Personality traits and RRS brooding at baseline

	**All**			**CBT**			**TAU**		
**T&P**	**r**	***p***	**q_FDR**	**r**	***p***	**q_FDR**	**r**	***p***	**q_FDR**
anxious worrying	0.47	<.001	0.008	0.42	.016	0.043	0.48	.001	0.004
personal reserve	0.38	<.001	0.008	0.40	.025	0.050	0.40	.008	0.016
rejection sensitivity	0.56	<.001	0.008	0.51	.003	0.024	0.58	<.001	0.007
perfectionism	0.19	.10	0.11	0.15	.42	0.48	0.20	.22	0.25
self-focused	0.087	.46	0.46	-0.47	.80	0.80	0.26	.10	0.13
social avoidance	0.21	.079	0.11	0.40	.025	0.05	0.57	.72	0.72
irritability	0.29	.013	0.021	0.25	.17	0.23	0.40	.008	0.016
self-criticism	0.47	<.001	0.008	0.47	.006	0.024	0.45	.003	0.008

S1B: Personality traits and RRS brooding at week 16

	**All**			**CBT**			**TAU**		
**T&P**	**r**	***p***	**q_FDR**	**r**	***p***	**q_FDR**	**r**	***p***	**q_FDR**
anxious worrying	0.15	0.21	0.42	-0.086	0.65	1.00	0.23	0.17	0.27
personal reserve	0.15	0.21	0.42	0.038	0.84	0.96	0.35	0.03	0.12
rejection sensitivity	0.33	0.006	0.048	0.13	0.50	1.00	0.45	0.005	0.040
perfectionism	-0.043	0.73	0.83	-0.17	0.36	0.96	0.009	0.96	0.96
self-focused	0.009	0.94	0.94	0.008	0.97	0.97	0.091	0.59	0.67
social avoidance	0.19	0.13	0.35	0.18	0.34	1.00	0.23	0.17	0.27

irritability	0.12	0.34	0.45	0.067	0.73	0.97	0.24	0.15	0.30
self-criticism	0.29	0.015	0.060	0.18	0.33	1.00	0.33	0.04	0.11

S1C: Personality traits and GRID-HAMD17 at baseline

	**All**			**CBT**			**TAU**		
**T&P**	**r**	***p***	**q_FDR**	**r**	***p***	**q_FDR**	**r**	***p***	**q_FDR**
anxious worrying	0.077	.51	0.58	0.13	.48	1.00	0.089	.57	0.76
personal reserve	0.28	.016	0.13	0.34	.057	0.23	0.27	.089	0.36
rejection sensitivity	0.23	.047	0.19	0.080	.66	1.00	0.33	.030	0.24
perfectionism	0.17	.15	0.30	0.47	.007	0.056	0.071	.66	0.75
self-focused	0.15	.22	0.35	−0.07	.70	0.70	0.25	.11	0.29
social avoidance	0.064	.59	0.59	0.10	.57	1.00	0.052	.74	0.74
irritability	0.18	.12	0.32	0.074	.69	0.92	0.24	.12	0.24
self-criticism	0.097	.41	0.55	0.074	.69	0.92	0.13	.41	0.66

S1D. Personality traits and GRID-HAMD17 at week 16

	**All**			**CBT**			**TAU**		
**T&P**	**r**	***p***	**q_FDR**	**r**	***p***	**q_FDR**	**r**	***p***	**q_FDR**
anxious worrying	-0.03	0.81	0.81	-0.1	0.59	0.67	-0.033	0.84	0.84
personal reserve	0.21	0.085	0.68	0.23	0.21	0.84	0.21	0.19	0.38
rejection sensitivity	0.098	0.42	0.84	-0.19	0.31	0.83	0.33	0.038	0.30
perfectionism	-0.035	0.78	0.89	-0.16	0.40	0.64	0.052	0.76	0.87
self-focused	-0.15	0.20	0.53	-0.19	0.32	0.64	-0.087	0.60	0.96
social avoidance	0.062	0.61	0.98	0.06	0.75	0.75	0.067	0.68	0.91
irritability	-0.062	0.61	0.98	-0.30	0.11	0.88	0.26	0.11	0.44
self-criticism	0.19	0.11	0.44	0.12	0.52	0.69	0.23	0.16	0.43
